# Polymorphisms of Phase I and Phase II Enzymes and Breast Cancer Risk

**DOI:** 10.3389/fgene.2012.00258

**Published:** 2012-11-28

**Authors:** Christina Justenhoven

**Affiliations:** ^1^Dr. Margarete Fischer-Bosch-Institute of Clinical PharmacologyStuttgart, Germany; ^2^University of TübingenTübingen, Germany

**Keywords:** breast cancer risk, tumor histo-pathology, phase I and II metabolism, polymorphisms, sequence homology

## Abstract

Breast cancer is a complex disease which is provoked by a multitude of exogenous and endogenous factors including genetic variations. Recent genome-wide association studies identified a set of more than 18 novel low penetrant susceptibility loci, however, a limitation of this powerful approach is the hampered analysis of polymorphisms in DNA sequences with a high degree of similarity to other genes or pseudo genes. Since this common feature affects the majority of the highly polymorphic genes encoding phase I and II enzymes the retrieval of specific genotype data requires adapted amplification methods. With regard to breast cancer these genes are of certain interest due to their involvement in the metabolism of carcinogens like exogenous genotoxic compounds or steroid hormones. The present review summarizes the observed effects of functional genetic variants of phase I and II enzymes in well designed case control studies to shed light on their contribution to breast cancer risk.

## Introduction

The implementation of cost effective high-throughput genotyping methods enables the determination of genotypes at large scale and fast pace. These improvements are prerequisite of the in depth investigation of the polygenetic basis of complex diseases. Prominent examples are genome-wide association studies which led to the identification of novel breast cancer risk factors such as polymorphisms in *FGFR2*, *CCND1*, *TOX3*, *MAP3K1*, *LSP1*, *CDKN2A*, and *2B* (Easton et al., 2007; Lambrechts et al., 2012). However, a shortcoming of this comprehensive approach is the exclusion of the majority of genes encoding phase I and II enzymes, because their special genomic architecture hampers the assessment of accurate genotype data. It is necessary to overcome this limitation due to the fact that functional genetic variations in these genes are known to alter expression, activity, and stability of the encoded enzymes causing defective inactivation and excretion of hormones as well as environmental toxicants (Thompson and Ambrosone, 2000; Reszka et al., 2006). Thus, it is of high relevance to understand the potential impact of these polymorphisms in pathogenic processes such as carcinogenesis. In addition, these phase I and II genes play a pivotal role in activation and metabolism of drugs with the potential to trigger therapy response as well as occurrence of adverse side effects (Meyer et al., 2012). With respect to breast cancer pharmacogenetic investigations revealed the impact of a genetical determined poor metabolizer phenotype of the phase I enzyme cytochrome P450 (CYP) 2D6 and tamoxifen treatment outcome (Schroth et al., 2009). This finding has been a matter of debate due to reports on conflicting results that seem to be based on inaccurate genotype data (Brauch et al., 2012). Amongst others this finding underlines the need of specific genotyping methodologies for genes encoding metabolic enzymes. This review will focus on studies investigating the role of genetic variants of phase I and II enzymes in breast cancer risk that used validated genotyping methods.

## Breast Cancer Risk

Breast cancer is a multifactorial disease and it is known that the carcinogenic process is affected by several endogenous as well as exogenous factors (Rebbeck et al., 1997). In this respect, steroid hormones play a pivotal role (Key et al., 2002b). Epidemiological studies indicated an increased breast cancer risk in women with prolonged exposure to sex hormones, e.g., early menarche and late menopause (Henderson and Feigelson, 2000; Clemons and Goss, 2001). Moreover, observational studies revealed the risk effect of exogenous hormones such as postmenopausal hormone replacement therapy (HRT; Rossouw et al., 2002; Beral and Million Women Study Collaborators, 2003; Pesch et al., 2005; Flesch-Janys et al., 2008) and oral contraceptives (Collaborative Group on Hormonal Factors in Breast Cancer, 1996; Kahlenborn et al., 2006). The strong correlation between circulation steroid hormones and breast cancer risk is supported by an observation of a two-fold increased risk for women with elevated sex hormone levels (Key et al., 2002a; Eliassen et al., 2006). A functional explanation of these findings comes from *in vitro* and *in vivo* studies that indicated initiation, promotion, and progression of breast tumorigenesis by estrogens and their metabolites (Nandi et al., 1995; Yue et al., 2003; Turan et al., 2004). This effect has been attributed to estrogen-induced gene expression of factors involved in cell growth and division (Liu and Lin, 2004) as well as genotoxic action of metabolic compounds such as 4-hydroxy catechol estrogens and estrogen-3,4-quinones (Yager and Davidson, 2006). Moreover, progesterone adds to hormone-induced carcinogenesis by promotion of estrogen synthesis, estrogen receptor expression, and cell proliferation (Poutanen et al., 1995; Shyamala et al., 2002; Moore et al., 2006; Pawlak and Wiebe, 2007). Beyond hormonal factors environmental carcinogens, e.g., tobacco smoke, or genetic factors, e.g., mutations and polymorphisms contribute to breast cancer susceptibility. A genetic basis of breast cancer has been suggested by family studies indicating a two-fold increased risk in the first-degree relatives of women with the disease (Collaborative Group on Hormonal Factors in Breast Cancer, 2001). In the 1990s, the two major breast cancer susceptibility genes *BRCA1* and *BRCA2* were identified (Miki et al., 1994; Wooster et al., 1995) revealing that harmful mutations in these genes confer to a cumulative disease risk by age 70 years of 65 and 45%, respectively (Antoniou et al., 2003). In the following years further genetic factors with different penetrance and frequency have been described. As of today less than 5% of familial breast cancer were attributed to high penetrance breast cancer genes *BRCA1*, *BRCA2*, *PTEN*, *MSH2*, *STK11*, *CDH1*, and *TP53* (Wooster and Weber, 2003; Malone et al., 2006; Walsh et al., 2006) and rare genetic variants at *ATM*, *CHEK2*, *BRIP*, *NBN*, *RAD50*, or *PALB2* that jointly confer an approximately two-fold increased risk (Meijers-Heijboer et al., 2002; The CHEK2 Breast Cancer Case-Control Consortium, 2004; Rahman et al., 2007). Recent genome-wide association studies revealed strong evidence for more than 18 common breast cancer susceptibility alleles including *FGFR2*, *CCND1*, *TNRC9*, *MAP3K1*, and *LSP1* (Cox et al., 2007; Easton et al., 2007; Lambrechts et al., 2012). Most of these genes are related to DNA repair, cell cycle control, apoptosis, cell growth, and division, representing the most important pathways for the protection of cells against carcinogenic processes. However, the lack of observed risk associations with phase I and II enzymes is potentially based on their exclusion from genome-wide association studies due to hampered assay design or poor quality data which is reflected by the low coverage of these genes in current genotyping arrays (Gamazon et al., 2012).

## The Role of Phase I and II Enzymes in Carcinogenesis

Phase I and II enzymes are of particular interest with respect to breast cancer due to their involvement in the metabolism of steroid hormones, chemical carcinogens, and other environmental toxicants (Thompson and Ambrosone, 2000; Reszka et al., 2006). In phase I reaction substrates usually undergo reduction, oxidation, or hydroxylation yielding more polar metabolites; the predominant mediators of this phase are cytochrome P450 (CYP) enzymes (Guengerich, 1999). In most cases phase I metabolism is followed by phase II conjugation reactions. During phase II exogenous or endogenous compounds or their phase I metabolites are conjugated to a more polar molecule, a process that usually produces inactive and water soluble compounds which can be easily excreted by urine or bile (Smith et al., 1994; Turesky, 2004). Conjugating enzymes include glutathione-S-transferases (GSTs), sulfotransferases (SULTs), uridine diphosphate-glucuronosyltransferases (UGTs), *N*-acetyltransferases (NATs), and Methyltransferases. The combined phase I and II metabolism is mainly a detoxification and elimination process, however, both phases bear the risk of formation of toxic and highly reactive compounds which can induce or promote serious health problems such as cancer (Smith et al., 1994; Windmill et al., 1997). Thus, altered activity of metabolic enzyme holds the potential to increase the exposure to carcinogenic compounds and consequently the risk of tumor formation (Brockstedt et al., 2002).

## Challenges of Genotyping

**Figure 1:**
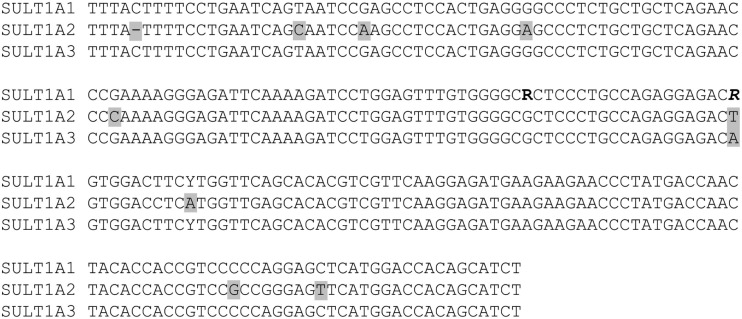
**Sequence homologies among the three members of the *SULT1A* gene subfamily located at chromosome 16 (NT_010393.16)**. A DNA fragment of 484 base pairs shows: the genetic variants *SULT1A1* 638 G > A (rs9282861, bold) and 667 A > G (rs1801030, bold/italic) as well as 100 base pairs upstream and downstream from these loci. Comparison of the DNA sequences shows that these genes differ only in a small of number of nucleotides (marked in gray) indicating sequence similarities of more than 90% between *SULT1A1*, *SULT1A2*, and *SULT1A3*.

**Figure 2:**
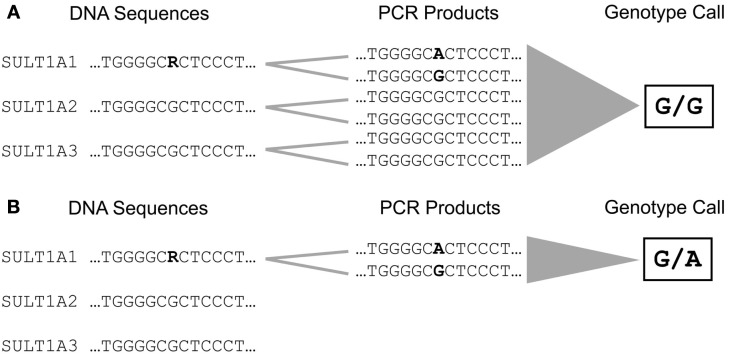
**Amplification and genotyping of the DNA sequence comprising the *SULT1A1* 638 G > A (rs9282861) polymorphism**. **(A)** The selection of unspecific primer binding sites lead to simultaneous amplification of *SULT1A1*, *SULT1A2*, and *SULT1A3* due to their high degree of sequence homology. This results in accumulation of amplification products carrying the referent G allele leading to an incorrect genotype call for rs9282861 (homozygous GG). **(B)** Selection of primer binding sites specific for *SULT1A1* enables amplification of the rs9282861 sequence region only resulting in correct determination of the genotype (heterozygous GA).

The majority of phase I and II enzymes are encoded by related genes which constitute gene families and subfamilies depending on their degree of sequence similarities. This particular genomic architecture hampers specific genotyping due to the potential co-amplification of homolog gene sequences. Therefore, the establishment of accurate analysis methods requires primer selection by eye inspection, adapted amplification protocols, and verification of genotype calls by an independent method (Justenhoven et al., 2010). An example for the particular need of an appropriate genotyping procedure is the analysis of the *SULT1A1* 638 G > A (rs9282861) polymorphism. The human *SULT1A* subfamily comprises three genes *SULT1A1*, *SULT1A2*, and *SULT1A3* which are located in close proximity on the short arm of chromosome 16 and share sequence similarities of more than 90% (Hempel et al., 2005). Due to these remarkable homologies the selection of applicable primers which enable specific amplification of the *SULT1A1* 638 G > A region is difficult (Figure 1). Usually automatic assay design tools generate inappropriate primers for such sequences which lead to simultaneous amplification of all members of a gene subfamily resulting in incorrect genotype calls due to abundance of the referent allele (Figure 2A). Valid assays include the identification of primer binding sites in unique DNA regions of the respective gene and adapted annealing temperatures, only such highly selective amplification conditions assure correct genotype calls (Figure 2B). Other gene families and subfamilies with a similar degree of sequence homologies are known for *CYP3A*, *CYP2C*, *GST*s, as well as *NAT*s and *UGT*s (Salinas and Wong, 1999; Gellner et al., 2001; Tukey and Strassburg, 2001; Nelson et al., 2004; Sim et al., 2008). So far individual assays for some of these polymorphisms have been established by researchers, e.g., for *CYP3A* (Justenhoven et al., 2010; The MARIE-GENICA Consortium on Genetic Susceptibility for Menopausal Hormone Therapy Related Breast Cancer Risk, 2010), *CYP2D6* (Schaeffeler et al., 2003; Morike et al., 2008), *CYP2C19* (Justenhoven et al., 2012), *GST*, *UGT*, and *SULT1A* (The MARIE-GENICA Consortium on Genetic Susceptibility for Menopausal Hormone Therapy Related Breast Cancer Risk, 2010) as well as companies (e.g., Applied Biosystems and Third Wave Technologies)^1^^,^^2^. Moreover, particular panels and arrays for the genetic analysis of metabolic enzymes and transporters have been developed within recent years: the AmpliChip^®^ CYP P450 Test^3^, the DMET Plus Panel DNA Chip^4^, VeraCode ADME Core Panel^5^, and the iPLEX ADME PGx Panel^6^. These tools were initially launched to support pharmacogenomic testing in clinical research and diagnostics, however, their coverage of relevant genes is still incomplete but they provide a convenient basis for a variety of investigations dealing with diverse health issues.

## Phase I and II Enzymes in Association with Breast Cancer Risk

Candidate gene approaches provide evidence for a particular role of metabolic enzymes in breast carcinogenesis. As of yet only a few studies analyzed the impact of polymorphisms in genes with high sequence homologies, whereas genes like *CYP1A1* and *CYP1B1* have been studied intensely (Economopoulos and Sergentanis, 2010; Sergentanis and Economopoulos, 2010). Therefore, this review focuses on those genes which are usually underrepresented in association studies due to technical issues. Literature search was done by PubMed^7^ using the key words “breast cancer polymorphism phase I,” “breast cancer polymorphism phase II,” “breast cancer polymorphism CYP” “breast cancer polymorphism UGT,” “breast cancer polymorphism SULT,” “breast cancer polymorphism GST,” and “breast cancer polymorphism NAT” in August 2012. In a next step studies analyzing associations between the respective polymorphisms and breast cancer risk factors or breast tumor characteristics were selected on the basis of study size, i.e., inclusion of more than 500 cases and 500 controls, DNA extracted from blood, validation of genotyping results by an independent method or meta analyses on summary data of at least five independent studies.

Significant associations, with *p* < 0.05 or 95% confidence interval not including 1.0, between polymorphic loci in genes encoding phase I and II enzymes and breast cancer risk are summarized in Table 1. It has been shown that functional genetic variants of the *CYP2C19* are associated with overall breast cancer risk and HRT-related breast cancer risk (Gan et al., 2011; Justenhoven et al., 2012). It is of note that these findings in two independent studies show similar effects. The variant CYP2C19*3 (rs57081121) which lead to a decreased activity of the CYP2C19 has been associated with increased risk in Asians (Gan et al., 2011) and the variant CYP2C19*17 (rs12248560) causing an ultra rapid metabolizer phenotype leads to a decreased HRT-related breast cancer risk in Europeans (Justenhoven et al., 2012). It is known that CYP2C19 catabolizes estrogens and progesterone (Yamazaki and Shimada, 1997; Cheng et al., 2001; Cribb et al., 2006) and the reported results suggest that increased metabolic activity of the CYP2C19 lowers endogenous hormone levels leading to a decreased risk.

**Table 1:** **Polymorphisms in phase I and II enzymes associated with breast cancer risk**.

Subgroup	Ethnicity	Gene	Polymorphism	Nucleotide exchange	Cases	Controls	Odds ratio	*p*-Value or 95% confidence interval	Reference
All	Asian	*CYP2C19*	rs57081121 (*3)	G > A	600	600	2.31	0.003	Gan et al. (2011)
	European	*UGT1A6*	rs6759892	T > G	3139	5466	1.17	0.014	The MARIE-GENICA Consortium on Genetic
									Susceptibility for Menopausal Hormone
									Therapy Related Breast Cancer Risk (2010)
	European	*UGT1A6*	rs2070959	A > G	3147	5484	1.22	0.007	The MARIE-GENICA Consortium on Genetic
									Susceptibility for Menopausal Hormone
									Therapy Related Breast Cancer Risk (2010)
	Mixed	*GSTM1*	gene deletion	ins > del	1052	1098	1.86	1.12–3.08	Steck et al. (2007)
		*GSTT1*	gene deletion	ins > del					
		*GSTP1*	rs1695	G > A					

Premenopausal	European	*CYP3A*	rs10235235	T > C	4436	16393	0.91	0.03	Johnson et al. (2012)
women	African-American	*GSTT1*	gene deletion	ins > del	541	635	4.07	1.12–14.8	Van Emburgh et al. (2008)

Postmenopausal women	Mixed	*SULT1A1*	rs9282861	G > A	4623	7642	1.28	0.019	Jiang et al. (2010)

Postmenopausal women with BMI > 25 kg/m^2^	Asian	*SULT1A1*	rs9282861	G > A	1102	1147	3.6	1.5–8.7	Yang et al. (2005)

≥10 years use of	European	*GSTT1*	gene deletion	del > ins	2939	5237	1.04	0.0001	The MARIE-GENICA Consortium on Genetic
hormone									Therapy Related Breast Cancer Risk (2010)
replacement therapy									Susceptibility for Menopausal Hormone
	European	*GSTP1*	rs947894	C > T	2963	5269	1.05	0.022	The MARIE-GENICA Consortium on Genetic Susceptibility for Menopausal Hormone Therapy Related Breast Cancer Risk (2010)
	European	*CYP2C19*	rs12248560 (*17)	C > T	861	741	0.71	0.001	Justenhoven et al. (2012)

Smoker	European	*GSTT1*	Gene deletion	del > ins	2370	2624	1.3	1.1–1.6	Terry and Goodman (2006)
	European	*GSTM1*	Gene deletion	ins > del	2815	3170	1.4	1.1–1.9	Terry and Goodman (2006)
	African-American	*GSTP1*	rs1138272	C > T	541	635	2.12	1.02–4,41	Van Emburgh et al. (2008)
	European	*NAT2*	rs1801280	T > C	4837	6017	1.5	1.2–1.8	Terry and Goodman (2006)
			rs1799929	C > T	
			rs1208	A > G	
			rs1041983	T > C	
			rs1799930	G > A	
			rs1799931 (*5, *6, *7)	G > A	

*Studies with more than 500 breast cancer cases and 500 controls were included*.

The polymorphism rs10235235 located the non-coding region of the *CYP3A* locus has been associated with breast cancer risk in premenopausal women (Johnson et al., 2012). It would be of particular interest to follow-up this finding in independent case control collection and functional studies to understand the observed effect of this variant, because other genetic polymorphisms with known functional consequence located in *CYP3A4*, *CYP3A5*, *CYP3A7*, and *CYP3A43* showed no association with breast cancer risk (The MARIE-GENICA Consortium on Genetic Susceptibility for Menopausal Hormone Therapy Related Breast Cancer Risk, 2010).

Two functional genetic variants rs6759892 and rs2070959 which are located in the *UGT1A6* have been suggested to affect overall breast cancer risk. These variants did not show any association with hormonal factors (The MARIE-GENICA Consortium on Genetic Susceptibility for Menopausal Hormone Therapy Related Breast Cancer Risk, 2010), therefore, the risk effect is may be based on the role of UGT1A6 in the metabolism of exogenous compounds such as potential carcinogenic drug and food ingredients (Harding et al., 1988; Bock and Kohle, 2005).

It has been reported that the deletion of the *GSTM1* and *GSTT1* gene as well as the variant allele of the *GSTP1* rs1695 polymorphism impact overall breast cancer risk (Steck et al., 2007). Subgroup analyses showed an association of the *GSTT1* gene deletion and the *GSTP1* rs947894 variant with HRT-related breast cancer susceptibility (The MARIE-GENICA Consortium on Genetic Susceptibility for Menopausal Hormone Therapy Related Breast Cancer Risk, 2010). Moreover, the *GSTT1* deletion seems to affect breast cancer risk in premenopausal women (Van Emburgh et al., 2008). These observed effects of *GST* variants on hormone-related tumorigenesis is may be based on decreased conjugation of genotoxic estrogen quinones leading to elevated levels of DNA damage (Strange et al., 2001; Hachey et al., 2003). In addition, the *GSTM1* and *GSTT1* deletion as well as the *GSTP1* rs1138272 variant, were suggested to affect tobacco smoke-related breast cancer risk (Terry and Goodman, 2006) pointing to the potentially critical role of GSTs in the elimination of exogenous carcinogenic compounds such as polycyclic aromatic hydrocarbons (Hayes and Pulford, 1995).

The *SULT1A1* rs9282861 polymorphism has been associated with breast cancer risk in postmenopausal women, in particular with BMI > 25 kg/m^2^, suggesting a modifying effect of the variant allele on endogenous sex hormone exposure (Yang et al., 2005; Jiang et al., 2010).

It has been reported that the variant *NAT2* alleles rs1801280, rs1799929, rs1208, rs1041983, rs1799930, and rs1799931 lead to an increased smoking-related breast cancer which supports the hypothesis that slow acetylators may suffer greater exposure to tobacco carcinogens (Terry and Goodman, 2006).

## Phase I and II Enzymes and Breast Tumor Characteristics

Only a few well designed studies investigated the association between phase I and II enzymes and histo-pathological characteristics of breast tumors (Table 2). One study reported an association between the rs61469810 polymorphism of *CYP3A43* (CYP3A43*2A) and poorly differentiated breast tumors which may be explained by a potential contribution of the variant allele to increased sex hormone levels (Justenhoven et al., 2010). Another investigation suggested that the rs1058930 polymorphism of *CYP2C8* (CYP2C8*4) affects lymph node status of breast cancer patients (Jernstrom et al., 2009). The variant allele is known to lower metabolic activity of the encoded enzyme, however, the authors stated that an impact of the CYP2C9*2 allele which is in linkage disequilibrium with CYP2C8*4 cannot be excluded (Jernstrom et al., 2009).

**Table 2:** **Polymorphisms in phase I and II enzymes associated with histo-pathological characteristics of breast tumor**.

Subgroup	Ethnicity	Gene	Polymorphism	Nucleotide exchange	Cases		Odds ratio	*p*-Value	Reference
Grading	Europeans	*CYP3A43*	rs61469810 (*2A)	ins > delA	G1: 78	G > 1:854	1.74	0.010	Justenhoven et al. (2010)
Node status	Europeans	*CYP2C8*	rs1058930 (*4)	G > C	N0:62	*N* > 0: 16	0.18	0.002	Jernstrom et al. (2009)

*Studies with more than 500 breast cancer cases and 500 controls were included*.

## Conclusion

Genetic variations of phase I and II enzymes alter their activity or protein biosynthesis leading to defective detoxification and elimination of carcinogenic compounds. Due to a high degree of DNA sequence similarity among genes of subfamilies accurate genotyping requires elaborated methods and exhaustive quality control. Until now a few well designed studies give insights into the effect of polymorphisms in metabolic enzymes on breast cancer risk and point to their crucial action in steroid hormone catabolism. These finding underline the pivotal role of sex hormones in the regulation of proliferation, differentiation, and apoptosis as critical pathways for onset and progression of breast cancer (Schindler et al., 1998; Gruber et al., 2002; Seeger et al., 2003; Gadducci et al., 2005). However, a usual short coming is the publication bias related to findings without significant effect. Taken together, the prediction of breast cancer risk on polymorphisms of phase I and II enzymes is in its initial stage and prospective studies including different ethnic groups are needed in order to achieve genotyping based reliable risk determination. Recent developments of gene panels and arrays provide the technical basis for further assessment of the impact of variations in metabolic genes as well as gene–gene and gene-exposure interactions. Overall, comprehensive investigations of multiple genetic, endogenous, and exogenous factors will promote the understanding of the molecular mechanisms of breast carcinogenesis and support the improvement of prevention strategies.

## Conflict of Interest Statement

The author declares that the research was conducted in the absence of any commercial or financial relationships that could be construed as a potential conflict of interest.

## Footnotes

^1^http://www.appliedbiosystems.com

^2^http://www.twt.com

^3^http://www.roche.com

^4^http://www.affimetrix.com

^5^http://www.illumina.com

^6^http://www.sequenom.com

^7^http://www.ncbi.nlm.nih.gov
